# Hinokitiol protects primary neuron cells against prion peptide-induced toxicity *via* autophagy flux regulated by hypoxia inducing factor-1

**DOI:** 10.18632/oncotarget.8670

**Published:** 2016-04-09

**Authors:** Ji-Hong Moon, Ju-Hee Lee, You-Jin Lee, Sang-Youel Park

**Affiliations:** ^1^ Biosafety Research Institute, College of Veterinary Medicine, Chonbuk National University, Iksan, Jeonbuk, South Korea

**Keywords:** hinokitiol, prion protein, autophagy, HIF-1, neurodegeneration, Gerotarget

## Abstract

Prion diseases are fatal neurodegenerative disorders that are derived from structural changes of the native PrPc. Recent studies indicated that hinokitiol induced autophagy known to major function that keeps cells alive under stressful conditions. We investigated whether hinokitiol induces autophagy and attenuates PrP (106-126)-induced neurotoxicity. We observed increase of LC3-II protein level, GFP-LC3 puncta by hinokitiol in neuronal cells. Addition to, electron microscopy showed that hinokitiol enhanced autophagic vacuoles in neuronal cells. We demonstrated that hinokitiol protects against PrP (106-126)-induced neurotoxicity *via* autophagy by using autophagy inhibitor, wortmannin and 3MA, and ATG5 small interfering RNA (siRNA). We checked hinokitiol activated the hypoxia-inducible factor-1α (HIF-1α) and identified that hinokitiol-induced HIF-1α regulated autophagy. Taken together, this study is the first report demonstrating that hinokitiol protected against prion protein-induced neurotoxicity *via* autophagy regulated by HIF-1α. We suggest that hinokitiol is a possible therapeutic strategy in neuronal disorders including prion disease.

## INTRODUCTION

The misfolding and aggregation of specific proteins is a common hallmark of many neurodegenerative disorders, including highly prevalent illnesses such as Alzheimer's disease and Parkinson's disease, as well as prion diseases [1, 2]. One of the fundamental events related to TSE pathogenesis is the refolding of a host-encoded glycoprotein, the prion protein (PrPc), into a protease-insensitive isoform (PrPsc) that aggregates into deposits. The conversion into PrPsc is driven by the transition of the PrPc's large N-terminal region from a random coil to a β-sheet structure, which dominates the α-helix content (43% *vs*. 30%)[3, 4]. These profound changes in PrPc physicochemical properties bring out a structural transition. While PrPc is soluble in non-denaturing detergents, PrPsc is not. PrPc is smoothly digested by proteases, whereas PrPsc is partially resistant [5]. Although the major role PrPsc plays in the origin and transmission of TSEs is well established, how the disease-specific prion protein exerts its harmful effects on neurons is unknown [6].

A synthetic peptide corresponding to amino acid residues 106-126 of human PrP, which forms fibrils *in vitro*, is toxic to cultured hippocampal neurons. PrP (106-126) possesses many of the pathogenic and physiologic properties of PrPsc, including the ability to induce apoptosis in hippocampal neurons and induce astrocyte proliferation [7]. Prion diseases are associated with misregulation of autophagy as shown by the formation of giant autophagic vacuoles in experimental scrapie in hamsters [8].

Autophagy is a conserved trafficking pathway that is highly correlated by environmental conditions [9]. Autophagy, a common morphological feature in dying cells, has more recently been thought to keep cells alive under stressful conditions[10]. There are three defined types of autophagy: macro-autophagy, micro-autophagy, and chaperone-mediated autophagy (CMA), all of which promote proteolytic degradation of intracellular components in the lysosome[11]. We focused on macro-autophagy in the present study. Upon autophagy induction, the phagophore expands and encloses a portion of cytoplasm resulting in the formation of a double-membraned structure called the autophagosome, which fuses with a lysosome for degradation[12]. Microtubule-associated protein light chain 3 (LC3) is localized, aggregating on the autophagosome, and is considered a marker of autophagy. LC3B undergoes lipidation and is recruited to the phagophore, where it correlates membrane elongation and closure [13]. LC3B then transforms from LC3B-I to LC3B-II during autophagosome formation[14]. Also involved in autophagy is P62, a multifunctional signaling molecule associated with a variety of cellular pathways. P62 is one of the best-known autophagic substrates and is extensively employed as an indicator of autophagic degradation [15]. SQSTM1/p62 can deliver ubiquitinylated cargo to the proteasome, though they are mainly degraded by autophagy [15, 16]. SQSTM1/p62 levels are generally inversely related to autophagic degradation, since the loss of Atg genes or factors required for autophagosome fusion with lysosomes all result in a marked increase in SQSTM1/p62-positive aggregates [17, 18]. By eliminating damaged intracellular organelles and aggregates, autophagy promotes cell surface antigen presentation and cellular senescence, protects against genome instability and prevents necrosis [11, 19]. Thus, autophagy has an essential role in preventing diseases such as neurodegeneration, cancer, cardiomyopathy, diabetes, liver disease, autoimmune diseases and infections [11, 20–22].

Hypoxia inducible factor-1 is a heterodimeric transcription factor that plays a pivotal role in regulating cellular oxygen homeostasis. It is composed of an oxygen-regulated HIF-1α subunit and a constitutively expressed HIF-1β subunit. Under hypoxic conditions, HIF-1α hydroxylation is inhibited, allowing its translocation into the nucleus where it binds to HIF-1β to form an active complex, HIF-1. HIF-1 then initiates the transcription of an array of target genes that are vital for cellular adaption to hypoxia [23]. Recently, many non-hypoxic stimuli, such as cytokines, free radicals, growth factors, and hormones, have been shown to activate HIF-1α under normoxic conditions. Based on these data, HIF-1α appears to have a neuroprotective effect in the ischemic brain [24].

Hinokitiol is a tropolone-related compound found in various natural sources such as the heartwood of several cupressaceous plants. Hinokitiol has been widely used as an antimicrobial agent in hair tonics, toothpastes, cosmetics, and food [25]. Jayakumar et al. suggested that hinokitiol treatment provides neuroprotection, improved recovery of infarcted tissue, and improved neurological outcomes in embolic stroke-induced ischemic rats [26]. In addition, some studies suggest that hinokitiol activates the HIF pathway [27, 28] and autophagy [29, 30]. Based on these studies, we investigated whether hinokitiol induces HIF-1α stabilization and autophagy and whether these pathways have apoptotic or protective effects. We report that hinokitiol enhances autophagy and protects against prion protein-induced neurotoxicity *via* HIF-1α stabilization.

## RESULTS

### Hinokitiol-attenuated, prion protein-induced cytotoxicity in neuronal cells

**Figure 1:**
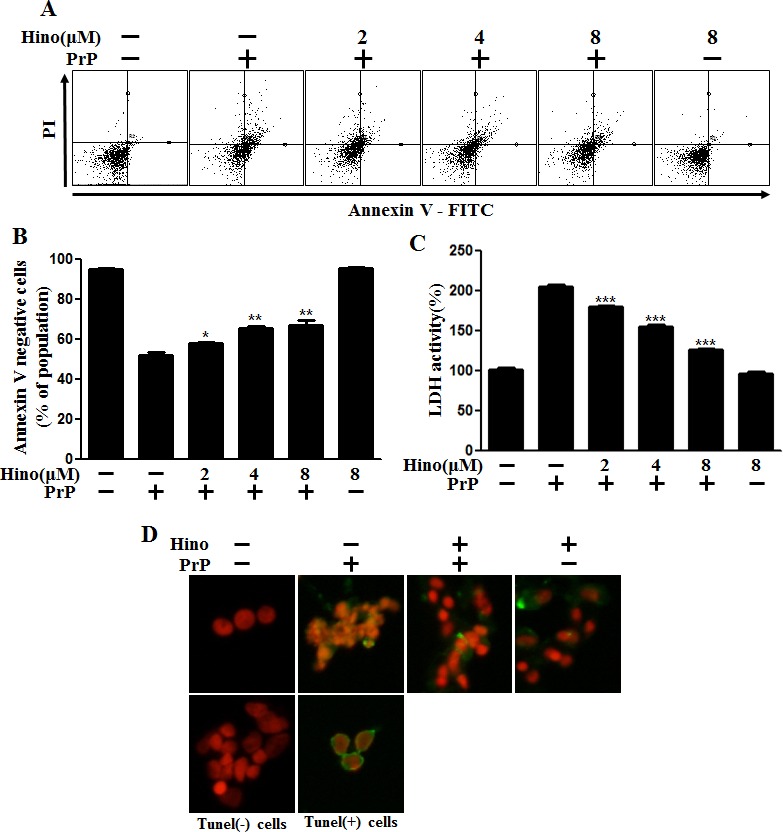
Hinokitiol attenuates PrP (106-126)-induced cytotoxicity in neuronal cells **A.** The primary neuronal cells were pretreated with hinokitiol (6 h) in a dose-dependent manner and then exposed to 100 μM PrP (106-126) for 12 h. Cell viability was measured by the annexin V assay. Cells were treated with FITC-annexin V, which binds to phosphatidylserine on the plasma membrane during apoptosis. **B.** Bar graph indicating the averages of the annexin V-negative cells. **C.** A lactate dehydrogenase (LDH) assay was used to quantify LDH released into the medium. **D.** Representative immunofluorescence images of TUNEL-positive (green) cells 12 h after exposure to 100 μM of PrP (106-126) in the absence or the presence of hinokitiol (6 h). The cells were counterstained with PI (red) to show all cell nuclei. *p < 0.05, **p < 0.01, *** *p* < 0.001: Significant differences between the control and treatment groups. Hino, hinokitiol; PrP, PrP (106-126).

We investigated the influence of hinokitiol on PrP (106-126)-induced neurotoxicity in primary neuron cells using an annexin V assay. The primary neurons were exposed to hinokitiol with or without PrP (106-126). The viability of PrP (106-126)-treated cells was decreased approximately 50% compared to controls, and hinokitiol-treated cells had enhanced viability. Cell viability in hinokitiol-treated only cells was comparable to untreated controls. Importantly, hinokitiol treatment inhibited PrP (106-126)-induced neurotoxicity in primary neurons (Figure 1A, 1B). LDH release levels indicate that hinokitiol inhibited PrP (106-126)-induced apoptosis in a dose-dependent manner (Figure 1C), consistent with previous results. As seen in Figure 1D, hinokitiol attenuated PrP (106-126)-induced apoptosis, which was evident based on the amount of DNA strand breakage.

### Hinokitiol-induced autophagy in neuronal cells

**Figure 2:**
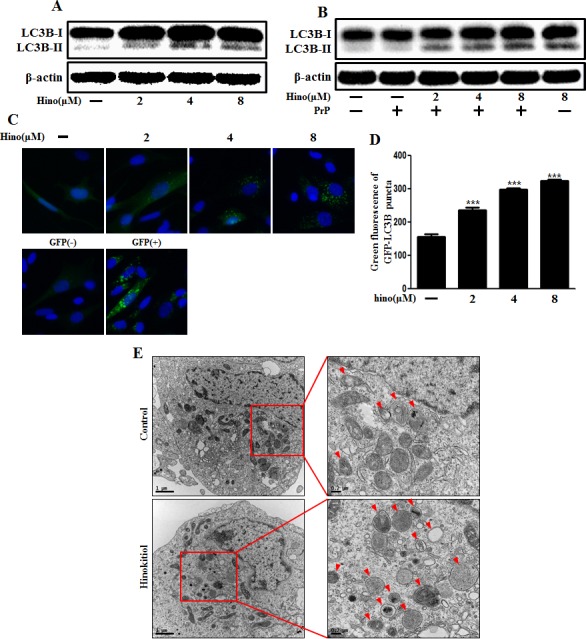
Hinokitiol induces autophagy in neuronal cells The primary neurons were treated with 2, 4, and 8 μM of hinokitiol for 6 h **A.** and were then exposed to 100 μM PrP (106-126) for 6 h **B.**. The treated cells were assessed for LC3B production by Western blot analysis. SK-N-SH cells were mixed with a titration (30MOI) of BacMam GFP-LC3B virus over 18 h and were then treated with hinokitiol in a dose-dependent manner for 6 h **C.**, **D.**. Negative control reagent and positive control reagent (CQ) at the same time. **E.** SK-N-SH cells were incubated with 8 μM of hinokitiol for 6 h and analyzed by TEM. Arrowheads indicate autophagosomes. *** *p* < 0.001; significant differences when compared with control and each treatment group. Hino, hinokitiol; PrP, Prion peptide (106-126); GFP (+), Positive control; GFP (−), Negative control.

We explored autophagy as a survival strategy for prion-induced neurotoxicity. First, we examined whether hinokitiol increased the autophagy marker, LC3B. LC3 protein is localized and aggregates on autophagosomes and is, therefore, considered a marker of autophagy. LC3 transforms from LC3-I to LC3-II during autophagosome formation [14]. We observed that levels of the late autophagosome marker LC3-II increased in the hinokitiol-treated group in a dose-dependent manner compared to the control and PrP (106-126)-treated groups *via* Western blot analysis in mouse primary neurons (Figure 2A, 2B). To visualize the activation of autophagy through the formation of autophagosomes in neurons, the Premo Autophagy Sensor (LC3B-FP) BacMam 2.0 system was employed. LC3B-FP and LC3B (G120A)-FP viral vectors (MOI = 30) were transduced into SK-N-SH cells, enabling the expression of fluorescent LC3B protein, and consequently, allowing us to monitor autophagosome dynamics using inverted fluorescent microscopy. Negative controls were established using mutant chimera LC3B (G120A)-FP. According to the results reported in Figure 2C and 2D, BacMam LC3B (G120A)-FP transduced cells showed a marked cytosolic and diffuse expression pattern. SK-N-SH cells treated with hinokitiol presented with an increased punctate fluorescent distribution pattern, suggesting LC3B-FP protein accumulation in the autophagosomes. We analyzed this reduction in LC3-II and green fluorescent puncta, which is caused by lysosomal autophagosome degradation. To detect further autophagic flux, transmission electron microscopy was performed. As shown in Figure 2E, double-membraned autophagosomes containing entrapped cytoplasm or entire organelles were induced by hinokitiol treatment. These results suggest that hinokitiol activates autophagy in human and mice neuronal cells.

**Figure 3:**
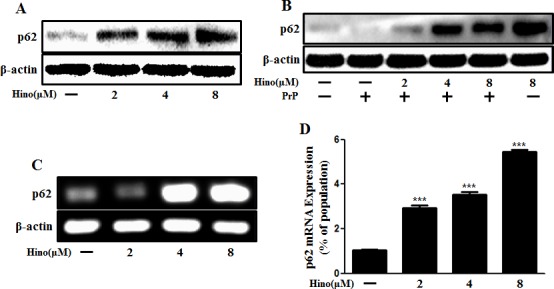
Hinokitiol increases p62/SQSTM1 mRNA and protein levels Primary neuronal cells were treated with hinokitiol in a dose-dependent manner for 6 h **A.** and were then exposed to 100 μM of PrP (106-126) for 6 h **B.**. The treated cells were assessed for p62/SQSTM1 expression by Western blot analysis. **C.** RT-PCR for the p62/SQSTM1 gene was analyzed from primary neuron cells. **D.** Real-time PCR for the p62/SQSTM1 gene was analyzed from primary neurons. *** *p* < 0.001; significant differences when compared with control and each treatment group. Hino, hinokitiol; PrP, Prion peptide (106-126).

According to our observations, SQSTM1/p62 protein was increased by hinokitiol treatment in neurons (Figure 3A, 3B). SQSTM1/p62 gene expression level was also increased with hinokitiol (Figure 3C, 3D). To determine the activity of the autophagic system during prion pathogenesis, alterations in the SQSTM1/p62 protein were assessed. The SQSTM1/p62 protein is a link between LC3 and ubiquitinated substrates and is incorporated into, and then degraded by, autolysosomes [31]. Usually, SQSTM1/p62 is degraded by autolysosomes when autophagy flux is activated. However, some studies suggest that p62 has a protective role and is required as a survival factor [32, 33]. In this case, we suggest that p62 is not involved in autophagy. By employing numerous experimental methods, we are confident that we have demonstrated autophagy.

### Hinokitiol inhibits PrP (106-126)-induced neuronal apoptosis by inducing autophagy

**Figure 4:**
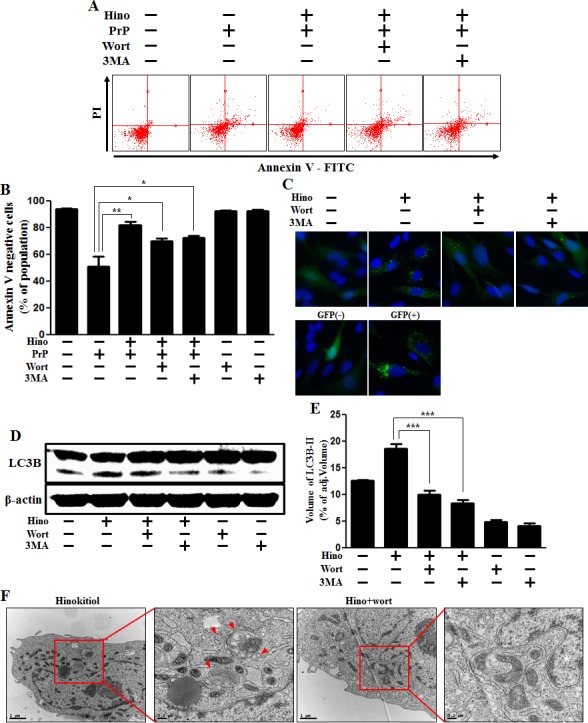
Hinokitiol-induced autophagy has a protective role in neuroblastoma cells **A.** The SK-N-SH neuroblastoma cells were pretreated with 8 μM of hinokitiol in the presence of autophagy inhibitors (3MA or wortmannin) for 6 hr and were then exposed to 100 μM of PrP (106-126) for 12 h. Cell viability was measured by the Annexin V assay. **B.** Bar graph indicating the averages of annexin V-negative cells. **C.** SK-N-SH cells were mixed with a titration (30MOI) of BacMam GFP-LC3B virus for 18 h and then treated with hinokitiol and autophagy inhibitors for 6h, Negative control reagent and positive control reagent (CQ) at the same time. **D.** Western blot for LC3B production was analyzed from SK-N-SH cells. Beta-actin was used as a loading control. **E.** Bar graph indicating the averages of LC3B-II expression levels. **F.** SK-N-SH cells were incubated with hinokitiol at 8 μM with wortmannin for 6 h and were then analyzed by TEM. Arrowheads indicate autophagosomes. *p < 0.05, **p < 0.01, *** *p* < 0.001; significant differences when compared with the control and each treatment group. Hino, hinokitiol; PrP, Prion peptide (106-126); wort, wortmannin; 3MA, 3-Methyladenine; GFP (+), Positive control; GFP (−), Negative control.

We recognize that the specific role of autophagy flux remains controversial. Therefore, we set out to determine whether autophagy flux has a protective function. We confirmed the effects of 3MA and wortmannin as autophagy inhibitors. We examined whether using these autophagy inhibitors could reduce the hinokitiol-induced neuroprotective effects against PrP (106-126). As shown in Figure 4A and 4B, the neuroprotective effects of hinokitiol diminished following treatment with the autophagy inhibitors. To visualize autophagy activation through the formation of autophagosomes, the Premo Autophagy Sensor (LC3B-FP) BacMam 2.0 system and Western blot analysis were employed as described above (Figure 4C, 4D, and 4E). We observed that the increase in LC3-II and green fluorescent puncta caused by the creation of autophagosomes was reduced with exposure to autophagy inhibitors. As shown in Figure 4D and 4E, LC-II protein expression level was decreased by the autophagy inhibitors. To confirm these results, transmission electron microscopy was performed (Figure 4F).

**Figure 5:**
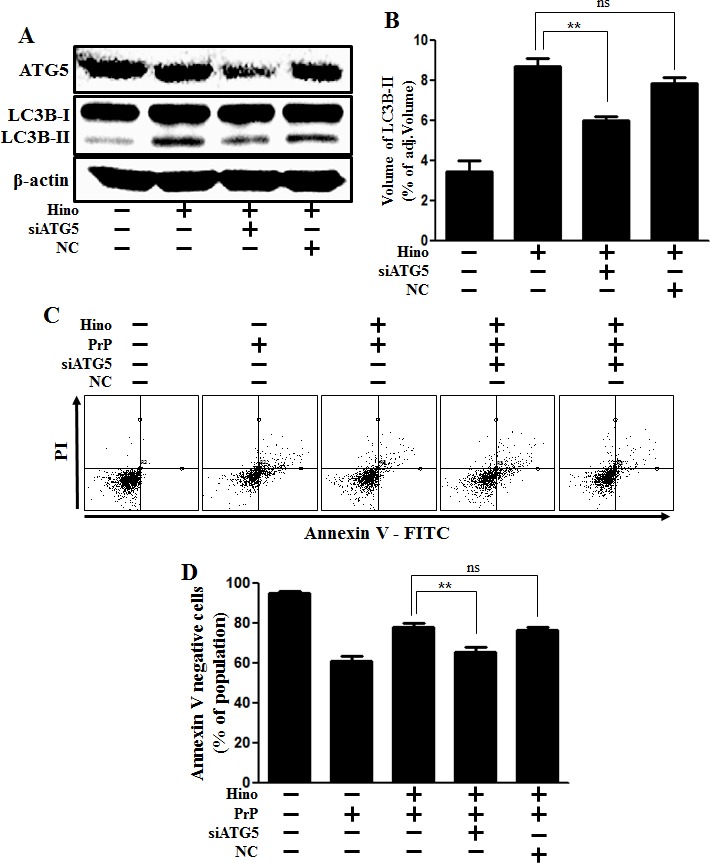
ATG5 knockdown decreases neuroblastoma cell viability **A.** ATG5 small interfering RNA (siATG5)- or negative control siRNA (NC)-transfected SK-N-SH cells were incubated with 8 μM of hinokitiol for 6 h. Western blot for ATG5 and LC3B production was analyzed in SK-N-SH cells. Beta-actin was used as a loading control. **B.** Bar graph indicating the averages of LC3B-II expression levels. **C.** SK-N-SH cells were incubated with PrP (106-126) for 12 h in the presence of hinokitiol. Cell viability was measured by Annexin V assay. **D.** Bar graph indicating the averages of annexin V-negative cells. **p < 0.01; significant differences when compared with control and each treatment group. Hino, hinokitiol; PrP, Prion peptide (106-126); NC, Negative control.

Additionally, knockdown of ATG5 using ATG5 small interfering RNA (ATG5 siRNA) decreased hinokitiol-induced autophagy (Figure 5A and 5B), as well as reduced the neuroprotective effects induced by hinokitiol in neuronal cells (Figure 5C and 5D).

### Autophagy induced by hinokitiol inhibits the PrP (106-126)-induced apoptotic pathway *via* HIF-1α stabilization

**Figure 6:**
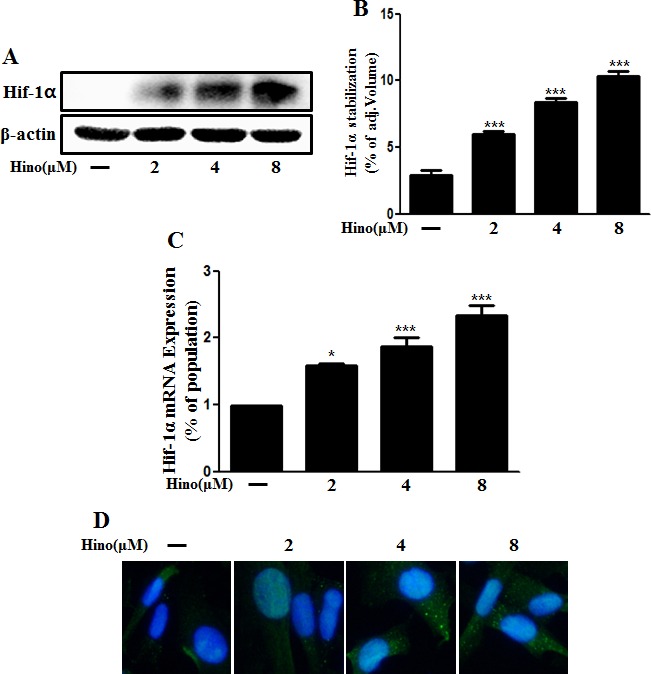
Hinokitiol induces Hif-1α stabilization **A.** The primary neurons were treated with 2, 4, and 8 μM of hinokitiol for 6 h. The treated cells were assessed for Hif-1α by Western blot analysis. **B.** Bar graph indicating the averages of Hif-1α stabilization levels. **C.** Real-time PCR for the Hif-1α gene was analyzed from primary neuron cells. **D.** Immunocytochemistry for Hif-1α protein was performed on SK-N-SH cells. *p < 0.05, *** *p* < 0.001; significant differences when compared with control and each treatment group. Hino, hinokitiol; Hif-1α, Hypoxia-inducible factor-1 alpha; p-Akt, phosphorylation of Akt; PrPc, prion protein.

Previously, it was suggested that hypoxia protects neuronal cells against PrP (106-126)-induced neurotoxicity and that this prevention is associated with hypoxia-mediated HIF-1α signaling [34]. Some reports suggest that hinokitiol activates the HIF pathway as described in the introduction. To assess expression of HIF-1α, primary neuronal cells were treated with hinokitiol, and both HIF-1α gene expression and protein levels increased in a dose-dependent manner (Figure 6A, 6B, and 6C). We confirmed this HIF-1α protein upregulation using immunocytochemistry (Figure 6D).

**Figure 7:**
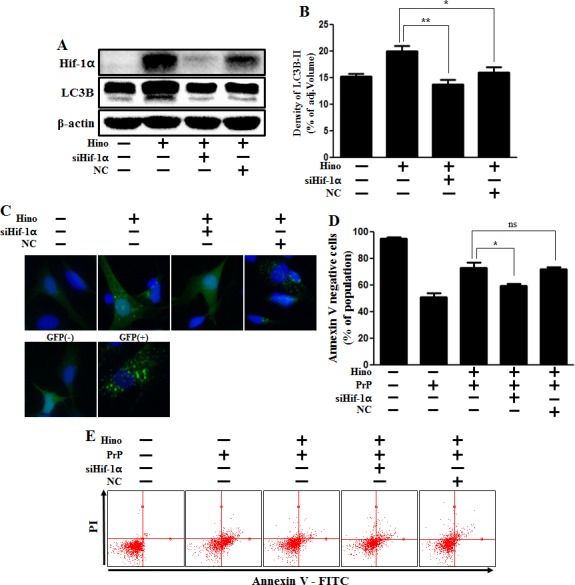
Hinokitiol-induced autophagy protects cytotoxicity *via* Hif-1α stabilization **A.** Hif-1α small interfering RNA (siHif-1α)-transfected or negative control siRNA (NC)-transfected SK-N-SH cells were incubated with 8 μM of hinokitiol for 6 h. Western blots for Hif-1α, LC3B proteins were analyzed from SK-N-SH cells. **B.** Bar graph indicating the averages of LC3B-II levels. **C.** SK-N-SH cells were mixed with a titration (30MOI) of BacMam GFP-LC3B virus over 18 h and were then treated with hinokitiol for 6 h, Negative control reagent and positive control reagent (CQ) at the same time. **D.** Bar graph of cell viability indicating the averages of annexin V-negative cells. **E.** Cell viability was measured by Annexin V assay from siRNA-treated cells. *p < 0.05; significant differences when compared with control and each treatment group. Hino, hinokitiol; Prion peptide (106-126); Hif-1α, Hypoxia-inducible factor-1 alpha; p-Akt, phosphorylation of Akt; NC, Negative control.

Furthermore, in order to confirm that hinokitiol protects against prion-induced apoptosis by HIF-1α stabilization, HIF-1α siRNA was used to knockdown HIF-1α gene expression. We found that knockdown of HIF-1α expression did block autophagy, as confirmed *via* Western blot (Figure 7A and 7B) and GFP-LC3 punta analysis (Figure 7C). In addition, we showed that knockdown of HIF-1α expression inhibited hinokitiol's neuroprotective effects (Figure 7D and 7E). We conclude that hinokitiol prevents prion-induced neurotoxicity by inducing autophagy and activating HIF-1α.

## DISCUSSION

This study demonstrates the possibility of attenuating prion protein-induced neurotoxicity with hinokitiol. It appears that hinokitiol attenuates neurotoxicity *via* autophagy, which is activated by HIF-1α stabilization. However, hinokitiol is not sufficient to prove a biological effect in neurons. We hope this study establishes a basis for further exploration of hinokitiol and its effects.

Autophagy is a dynamic lysosome-mediated process that involves the sequestration and delivery of cytoplasmic material to the lysosome where it is degraded and recycled [35, 36]. Several studies have proposed that autophagy is a double-edged sword, with both beneficial and harmful potential in cancer[37] and neurodegeneration[38]. Autophagy has several possible pathways depending on the circumstances, and the biochemical basis for its diverse functions is not well understood [39, 40]. Our results indicate that LC3-II was degraded by prion peptide treatment. Mizushima *et al*. suggested that LC3-II, which increases transiently upon induction of autophagy, is reduced after longer periods of autophagy activation [41]. For this reason, we suggest that degradation of LC3 indicates induction of autophagic flux.

Studies employing knockout, transgenic, and knock-in mice have shown that p62 plays a critical role in a number of cellular functions, including bone remodeling, obesity, and cancer [33, 42–44]. Recent findings link p62 activity to the extrinsic apoptosis pathway, and Mathew et al. showed that the modulation of p62 by autophagy is a key factor in tumorigenesis [45]. Previously, it was thought that p62 always has to be degraded when autophagy flux was induced. However, many reports suggest that p62 has various functions and correlates many signaling pathways. For example, upregulation of p62 may signify an inhibition of autophagic flux [46, 47]. In contrast, some studies report that the absence of p62 results in enhanced ROS production, a critical step for the induction of apoptosis [33]. Additionally, p62 depletion increases cell injury in neonatal rat ventricular myocytes (NRVMs) under basal conditions and during overexpression of misfolded proteins [48]. Haqa et al. suggested that mutant huntingtin can also form aggregates in the absence of p62, so they believe that the protective role of p62 may be to recruit autophagosomal components to the polyubiquitinylated protein aggregates rather than to facilitate the formation of these aggregates [32]. For these reasons, we believe that the increase in p62 in our case has a protective role in neuronal cells and does not influence autophagy. In addition, we checked increase of mRNA expression of p62. On the basis of our results and many studies, we suggest that it didn't appear p62 degradation in spite of autolysosome degradation in autophagy flux because protein of p62 was more synthesized than degradation.

Based on our results, we propose that hinokitiol may have a critical role as a therapeutic target for prion disease. We demonstrate that hinokitiol induces autophagy *via* the HIF-1α pathway, which acts as the main neuroprotective mechanism against prion peptide-induced neurotoxicity. The prion peptide 106-126 sequence is a useful model for *in vitro* study of prion-induced cell death, in addition to *in vivo* retinal neuron models treated with intravitreous injection of PrP fragments [49, 50]. In the future, we will further study the neuroprotective effects of hinokitiol, autophagy and the HIF-1α pathway in mouse models to examine hinokitiol's potential therapeutic role in prion disease.

## MATERIALS AND METHODS

### Cell culture

The primary neurons were isolated from embryonic day 18 mice. Briefly, tissues were collected in Hanks buffered saline solution without Mg2+ or Ca2+ (HBSS: Invitrogen-GIBCO, Grand Island, NY, USA), and digested in 0.25% trypsin with DNAse I (2000 units/mg) (Invitrogen, Carlsbad, CA, USA) for 20 min at 37°C. Cells were then mechanically dissociated and diluted in HBSS containing Mg2+ and Ca2+. Isolated neuron cells were diluted in DMEM containing 25 mM glucose (abbreviated in text as glucose medium; Thermo Scientific) supplemented with 10% FBS and were plated in flasks pre-coated with 50 μg/ml poly D-lysine. The human neuroblastoma cell line SK-N-SH was obtained from the American Type Culture Collection (ATCC, Rockville, MD, USA). Cells were cultured in minimum essential medium (MEM, Hyclone Laboratories, Logan, UT, USA) containing 10% fetal bovine serum (Invitrogen-GIBCO, Grand Island, NY, USA) and gentamycin (0.1 mg/mL) in a humidified incubator maintained at 37°C and 5% CO_2_.

### PrP (106-126) treatment

Synthetic PrP (106-126) peptides (sequence: Lys-Thr-Asn-Met-Lys-His-Met-Ala-Gly-Ala-Ala-Ala-Ala-Gly-Ala-Val-Val-Gly-Gly-Leu-Gly) were synthesized by Peptron (Seoul, Korea). The peptides were dissolved in sterile dimethyl sulfoxide at a stock concentration of 10 mM and stored at −20°C.

### Annexin V assay

Apoptosis in detached cells was assessed using an annexin V assay kit (Santa Cruz Biotechnology, Santa Cruz, CA, USA) according to the manufacturer's protocol. Annexin V levels were determined by measuring fluorescence at 488 nm of excitation and 525/30 emission using a Guava easyCyte HT System (Millipore, Bedford, MA, USA).

### Terminal deoxynucleotidyl transferase dUTP nick end labeling (TUNEL) assay

TUNEL analysis was performed to measure the degree of cellular apoptosis using an *in situ* ApoBrdU DNA fragmentation assay kit (BioVision, Mountain View, CA, USA) following the manufacturer's instructions. Cells were counterstained with propidium iodide (PI) to show cell nuclei.

### Lactate dehydrogenase assay

Cytotoxicity was assessed in the supernatants using a lactate dehydrogenase (LDH) cytotoxicity detection kit (Takara Bio, Inc., Tokyo, Japan) according to the manufacturer's protocol. LDH activity was determined by measuring absorbance at 490 nm using a microplate reader (Spectra Max M2, Molecular Devices, Sunnyvale, CA, USA).

### BacMam transduction

Wild-type or mutant GFP-tagged LC3B was expressed in cells by adding the appropriate concentrations of the appropriate virus from the Premo Autophagy Sensor LC3B-GFP kit (BacMam 2.0) (Life Technologies P36235) to the growth medium as indicated in the figure legends.

### Immunocytochemistry

Immunocytochemical analyses were performed on neuroblastoma cells with anti-p62 (P0067, Sigma Aldrich) antibodies. Cells were cultured on glass slides (Nalge Nunc International, Naperville, IL). Cells were washed in sterilized TBST for 10 min, then blocked for 15 min with 5% FBS in TBST, and then incubated overnight at 4°C with the primary antibodies diluted with 5% FBS in TBST. Alexa Fluor 488-labeled donkey anti-rabbit IgG antibody diluted to 1:1000 (Molecular Probes, A21206) was used to visualize channel expression using fluorescence microscopy.

### RNA interference

SK-N-SH cells were transfected with ATG5 small interfering RNA (siRNA: oligoID HSS114104: Invitrogen, Carlsbad, CA, USA) and HIF-1α siRNA (oligoID HSS104775: Invitrogen) using Lipofectamine 2000 according to the manufacturer's instructions. After a 48-hr culture, knockdown efficiency was measured at the protein level by immunoblot analysis. Nonspecific siRNA (oligoID 12935-300: Invitrogen) was used as the negative control.

### Western blot analysis

Primary neuronal cells and SK-N-SH cells were placed in lysis buffer [25 mM HEPES (4-(2-hydroxyethyl)-1-piperazineethanesulfonic acid), pH 7.4, 100 mM NaCl, 1 mM EDTA (ethylene diamine tetra acetic acid), 5 mM MgCl_2_, 0.1 mM DTT (dithiothreitol), and a protease inhibitor mixture]. Whole cell proteins were electrophoresed on a 10-15% sodium dodecyl sulfate polyacrylamide gel and transferred to a nitrocellulose membrane. Immunoreactivity was detected through sequential incubation with primary antibodies, horseradish peroxidase-conjugated secondary antibodies, and an enhanced chemiluminescence reagent, *i.e.,* West save gold detection kit (AbFrontier Inc.). The primary antibodies used for immunoblotting were anti-LC3B (#4108, Cell Signaling Technology), anti-P62 (#5114, Cell Signaling Technology), anti-ATG5 (#2630, Cell Signaling Technology) and anti-β-actin (A5441, Sigma Aldrich). Images were examined using a Fusion FX7 imaging system (Vilber Lourmat, Torcy Z.I., Sud, France). Signal band densitometry was analyzed using the Bio-1D software (Vilber Lourmat, Marne La Vallee, France).

### Quantitative real-time polymerase chain reaction (qRT-PCR)

Total ribonucleic acid (RNA) was extracted from primary neuronal cells using the easy-spin™ Total RNA Extraction Kit (Intron Biotechnology, Seoul, Korea). The cDNA synthesis was carried out following the instructions in the TaKaRa PrimeScript TM 1st strand cDNA synthesis kit (TaKaRa Bio, Tokyo, Japan). For qRT-PCR, 1 μl of gene primers with SYBR Green (Bio-Rad Laboratories, Hercules, CA, USA) in 20 μl of reaction volume was applied. The primers were: p62/SQSTM1 (forward: 5′CTCCCCAGACTACGACTTGTGT3′, reverse: 5′TCAACTTCAATGCCCAGAGG3′), Hif-1α (forward: 5′CGCAAGTCCTCAAAGCACAGTTAC3′, reverse: 5′TGGTAGTGGTGGCATTAGCAGTAG3′), and β-actin (as an internal control) (forward: 5′GCAAGCAGGAGTATGACGAG3′, reverse: 5′CAAATAAAGCCATGCCAATC3′). All reactions with iTaq SYBR Green Supermix (Bio-Rad Laboratories, Hercules, CA, USA) were performed on the CFX96 real-time PCR detection system (Bio-Rad Laboratories, Hercules, CA, USA).

### Transmission electron microscopy analysis

TEM samples were analyzed by Transmission Electron Microscope (JEM-2010, JEOL) installed in the Center for University-Wide Research Facilities (CURF) at Chonbuk National University. After fixing SK-N-SH cell samples in 2.5% glutaraldehyde (TED PELLA, USA) in PBS (pH: 7.2), specimens were post-fixed in 1% osmium tetroxide (Heraeus, South Africa), dehydrated in graded ethanol and propylene oxide (Acros Organics, USA), and then embedded in epoxy resin (Embed812, NMA: Nadic methyl anhydride, DDSA: Dodenyl Succinic Anhidride, DMP-30, USA), as used previously. Serial ultrathin sections were cut on an LKB-III ultratome (LEICA, Germany). Ultrathin sections were stained with uranyl acetate (TED PELLA, USA) and lead citrate (TED PELLA, USA) and were examined using a Hitachi H7600 electron microscope (Hitachi, Japan) at an accelerating voltage of 100 kV.

### Statistical analysis

Unpaired *t*-tests or Welch's corrections were used to compare differences between the two groups. One-way ANOVAs followed by the Dunnett's test were used for multiple comparisons. All statistical analyses were performed with GraphPad Prism software. Results were considered significant at * *p* < 0.05, ** *p* < 0.01 or *** *p* < 0.001.
